# Supplementary material for: Low dose ionizing radiation strongly stimulates insertional mutagenesis in a γH2AX dependent manner
Source: PLoS Genet. 2020 Jan 16;16(1):e1008550. doi: 10.1371/journal.pgen.1008550 (PMC6964834; doi:10.1371/journal.pgen.1008550)
Supplement: S1 Fig — (A) S-RI in HeLa cells transfected by electroporation with linear or circular plasmid DNA carrying puromycin resistance gene under a PGK promoter. (B) S-RI in HeLa cells transfected by lipofection. (C) Representative FACS plots from the rAAV S-RI experiments shown in (Fig 1D). HeLa cells infected with rAAV2-GFP virus and analyzed at different time points after infection. Gates P4 and P7 were used to calculate the fraction of high GFP-positive and all GFP-positive cells, respectively. The latter better reflects transient transduction efficiency, while the former more reliably represents the population of cells that still continuously produce the GFP protein. Percentage of GFP-positive cells drops precipitously from day 2 to day 8. At 8 days post infection the broader gate (P7) still contains cells that express GFP from transient infection, while the more stringent gate contains cells stably expressing GFP. (D) FACS-based S-RI assay performed with U2OS cells electroporated with the GFP-encoding plasmid DNA. (E) Stimulation of RI in HeLa cells electroporated with circular or linear plasmid DNA and irradiated with 0.2 Gy at different time points after electroporation. (F) Stimulation of RI in mES cells electroporated with circular or linear plasmid DNA and irradiated with 1 Gy at different time points after electroporation. Colony numbers were adjusted for reduced viability due to irradiation, and normalized to unirradiated control. (G) Repeat of the experiment shown in (Fig 1H). (H) Background RI frequency (number of puromycin-resistant colonies per viable cell plated) from the experiments reported in (Fig 1H, S1G Fig). (PDF) [file pgen.1008550.s001.pdf]

Supplementary Figure S1

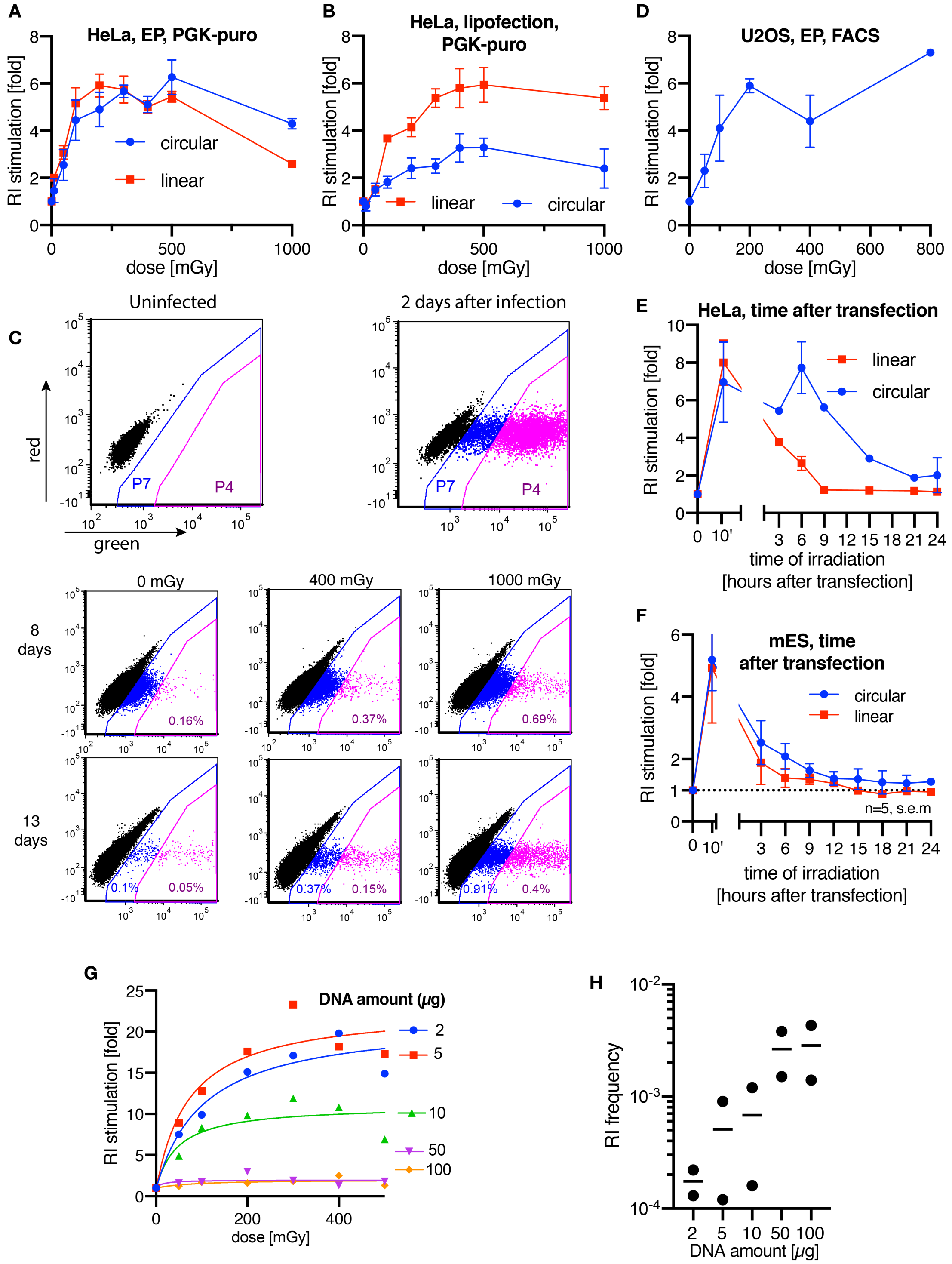

Figure S1. (related to Figure 1) (**A**) S-RI in HeLa cells transfected by electroporation with linear or circular plasmid DNA carrying puromycin resistance gene under a PGK promoter. (**B**) S-RI in HeLa cells transfected by lipofection. (**C**) Representative FACS plots from the rAAV S-RI experiments shown in **(Fig 1D)**. HeLa cells infected with rAAV2-GFP virus and analyzed at different time points after infection. Gates P4 and P7 were used to calculate the fraction of high GFP-positive and all GFP-positive cells, respectively. The latter better reflects transient transduction efficiency, while the former more reliably represents the population of cells that still continuously produce the GFP protein. Percentage of GFP-positive cells drops precipitously from day 2 to day 8. At 8 days post infection the broader gate (P7) still contains cells that express GFP from transient infection, while the more stringent gate contains cells stably expressing GFP. (**D**) FACS-based S-RI assay performed with U2OS cells electroporated with the GFP-encoding plasmid DNA. (**E**) Stimulation of RI in HeLa cells electroporated with circular or linear plasmid DNA and irradiated with 0.2 Gy at different time points after electroporation. (**F**) Stimulation of RI in mES cells electroporated with circular or linear plasmid DNA and irradiated with 1 Gy at different time points after electroporation. Colony numbers were adjusted for reduced viability due to irradiation, and normalized to unirradiated control. (**G**) Repeat of the experiment shown in **(Fig 1H)**. (**H**) Background RI frequency (number of puromycin-resistant colonies per viable cell plated) from the experiments reported in **(Fig 1H, S1G)**.
