# Supplementary material for: Low dose ionizing radiation strongly stimulates insertional mutagenesis in a γH2AX dependent manner
Source: PLoS Genet. 2020 Jan 16;16(1):e1008550. doi: 10.1371/journal.pgen.1008550 (PMC6964834; doi:10.1371/journal.pgen.1008550)
Supplement: S2 Fig — (A) Background RI in the mutant cell lines used in S-RI assay from (Fig 2A). Individual values from biological replicas are plotted, with bars indicating means ±s.e.m. Statistical significance was determined using one-way ANOVA with Dunnett’s multiple comparison test. (B) Immunoblot of H2ax-/- and complemented lines. Total cell lysates from wild-type, H2ax-/- (A) and (N) lines, and H2ax-/- (N) line complemented with various H2AX mutants, were immunoblotted with the indicated antibodies. To test γH2AX induction cells were irradiated with 4 Gy and lysed 30 minutes after. (C) Background RI measured as in panel (A) in cells used in (Fig 2C). (D) Samples from plates used in the S-RI assay plotted in (Fig 2D) were taken to determine the effect of irradiation on clonogenic survival of the wild-type and H2ax-/- cells to compensate for loss of viability in S-RI. (E) Effect of DNA damage response kinase inhibitors on S-RI in wild-type (black circles) and DNA-PKcs-/- (red squares) mES cells. Cells were electroporated with linearized plasmid, seeded into dishes containing the indicated concentrations of the inhibitors and irradiated with 50 mGy. The chemicals were removed 6 hours later. Data from four independent experiments is plotted. (F) Cells were treated with the compounds used in the experiment shown in panel (E), irradiated with 0 to 10 Gy, lysed 4 h later, and analyzed by immunoblotting with the indicated antibodies. Phosphorylated and unphosphorylated forms of Chk2 are indicated with arrows on the upper blot. (PDF) [file pgen.1008550.s002.pdf]

Supplementary Figure S2

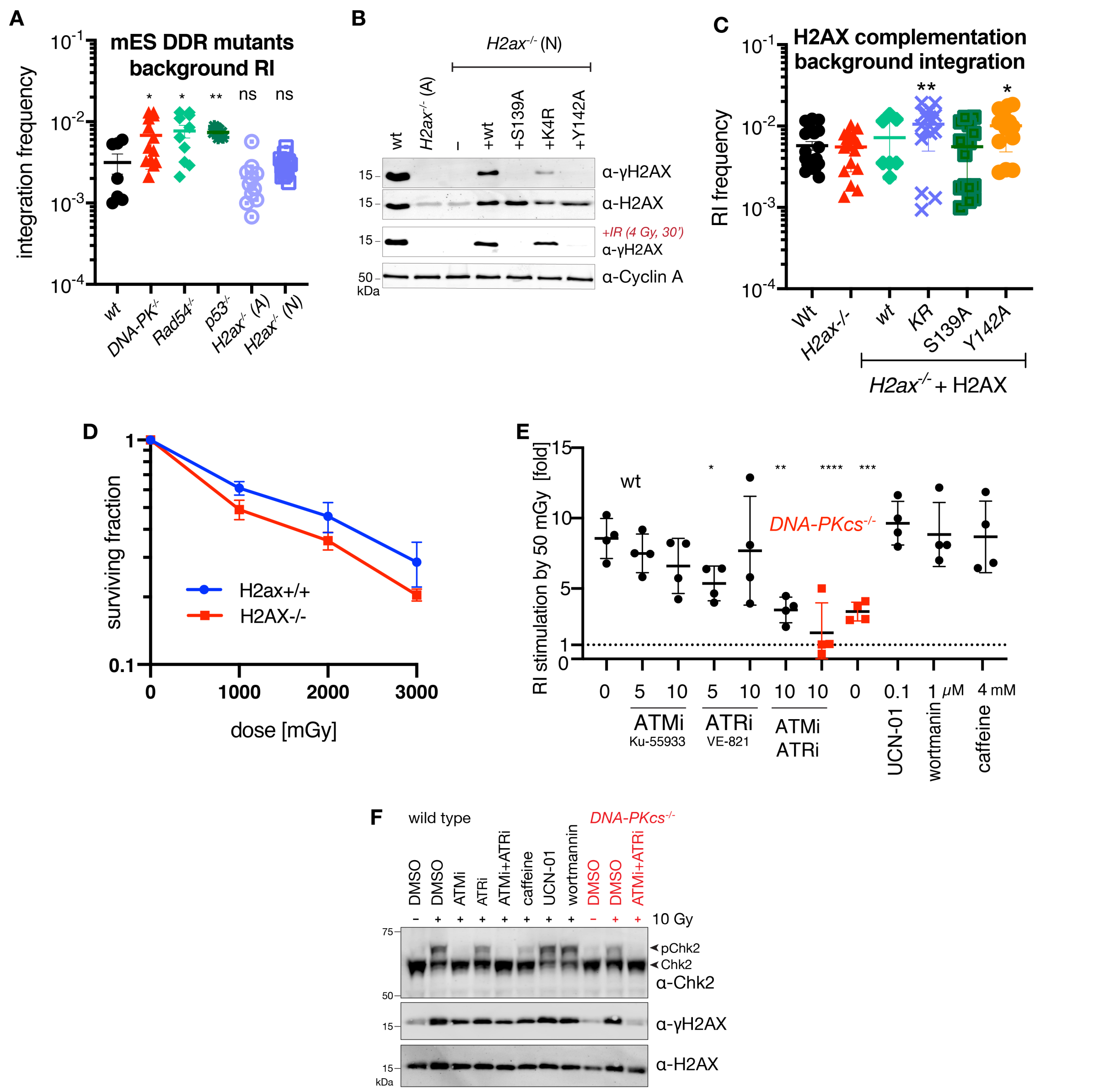

Fig S2. Genetic dependencies of stimulated random integration (related to Figure 2) (**A**) Background RI in the mutant cell lines used in S-RI assay from **(Fig 2A)**. Individual values from biological replicas are plotted, with bars indicating means ±s.e.m. Statistical significance was determined using one-way ANOVA with Dunnett's multiple comparison test. (**B**) Immunoblot of *H2ax*$^{-/-}$ and complemented lines. Total cell lysates from wild-type, *H2ax*$^{-/-}$ (A) and (N) lines, and *H2ax*$^{-/-}$ (N) line complemented with various H2AX mutants, were immunoblotted with the indicated antibodies. To test γH2AX induction cells were irradiated with 4 Gy and lysed 30 minutes after. (**C**) Background RI measured as in panel (A) in cells used in **(Fig 2C)**. (**D**) Samples from plates used in the S-RI assay plotted in **(Fig 2D)** were taken to determine the effect of irradiation on clonogenic survival of the wild-type and *H2ax*$^{-/-}$ cells to compensate for loss of viability in S-RI. (**E**) Effect of DNA damage response kinase inhibitors on S-RI in wild-type (black circles) and *DNA-PKcs*$^{-/-}$ (red squares) mES cells. Cells were electroporated with linearized plasmid, seeded into dishes containing the indicated concentrations of the inhibitors and irradiated with 50 mGy. The chemicals were removed 6 hours later. Data from four independent experiments is plotted. (**F**) Cells were treated with the compounds used in the experiment shown in panel (E), irradiated with 0 to 10 Gy, lysed 4 h later, and analyzed by immunoblotting with the indicated antibodies. Phosphorylated and unphosphorylated forms of Chk2 are indicated with arrows on the upper blot.
