# Supplementary material for: Low dose ionizing radiation strongly stimulates insertional mutagenesis in a γH2AX dependent manner
Source: PLoS Genet. 2020 Jan 16;16(1):e1008550. doi: 10.1371/journal.pgen.1008550 (PMC6964834; doi:10.1371/journal.pgen.1008550)
Supplement: S3 Fig — (A) 53BP1 knock-down does not affect S-RI efficiency. Means of four independent puromycin-resistant colony formation S-RI assays (two with linearized, two with circular plasmid DNA) are plotted. Immunoblot on total cell lysates with the indicated antibodies confirming the efficiency of knock-down is shown as an inset. (B) Background RI efficiency in mES cell lines deficient for γH2AX interacting proteins. Data is plotted as in (S2A Fig). (C) A model of RI and S-RI, based on the supposition that the initial stages of the two processes are mechanistically distinct ❶, to account for the observation that S-RI is γH2AX-dependent ❷, while RI is not ❸; MCPH1 competes with MDC1 for γH2AX binding, and its removal results in elevated RI ❸. MDC1 contributes to both RI and S-RI ❹, and 53BP1 can provide a backup mechanism for S-RI in the absence of MDC1, but does not contribute to the RI process ❹. Since neither RI nor S-RI are completely abolished by the deletion of the proteins listed in the scheme, alternative pathways must exist ❺. The final ligation steps are mediated by Pol θ or cNHEJ ❻, as we previously showed that all integration events in ES cells are abolished when both these end joining mechanisms are inactivated, however the relative contribution of Pol θ and cNHEJ to RI and S-RI may be different. (PDF) [file pgen.1008550.s003.pdf]

Supplmenetary Figure S3

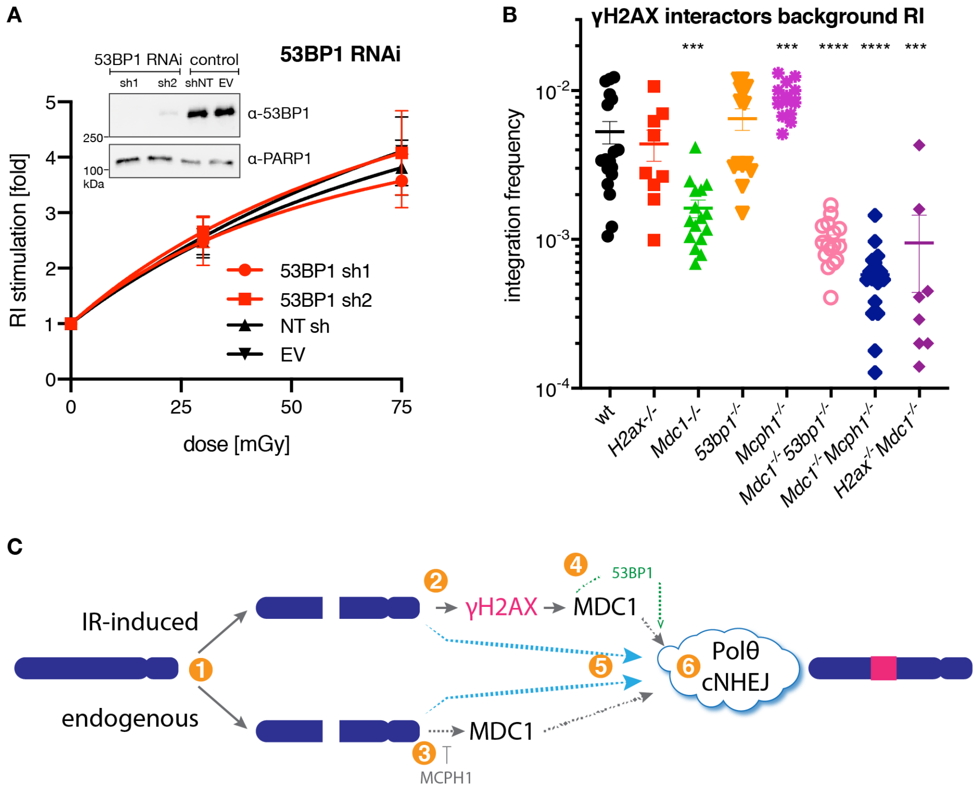

Figure S3 (related to Figure 3) (**A**) 53BP1 knock-down does not affect S-RI efficiency. Means of four independent puromycin-resistant colony formation S-RI assays (two with linearized, two with circular plasmid DNA) are plotted. Immunoblot on total cell lysates with the indicated antibodies confirming the efficiency of knock-down is shown as an inset. (**B**) Background RI efficiency in mES cell lines deficient for γH2AX interacting proteins. Data is plotted as in **(Fig S2A)**. (**C**) A model of RI and S-RI, based on the supposition that the initial stages of the two processes are mechanistically distinct ①, to account for the observation that S-RI is γH2AX-dependent ②, while RI is not ③; MCPH1 competes with MDC1 for γH2AX binding, and its removal results in elevated RI ③. MDC1 contributes to both RI and S-RI ④, and 53BP1 can provide a backup mechanism for S-RI in the absence of MDC1, but does not contribute to the RI process ④. Since neither RI nor S-RI are completely abolished by the deletion of the proteins listed in the scheme, alternative pathways must exist ⑤. The final ligation steps are mediated by Pol θ or cNHEJ ⑥, as we previously showed that all integration events in ES cells are abolished when both these end joining mechanisms are inactivated, however the relative contribution of Pol θ and cNHEJ to RI and S-RI may be different.
