# Supplementary material for: Low dose ionizing radiation strongly stimulates insertional mutagenesis in a γH2AX dependent manner
Source: PLoS Genet. 2020 Jan 16;16(1):e1008550. doi: 10.1371/journal.pgen.1008550 (PMC6964834; doi:10.1371/journal.pgen.1008550)
Supplement: S4 Fig — Schemes of mouse loci (A) Mdc1, (B) 53bp1 and (C) Mcph1 and of the corresponding gene targeting construct are shown. CRISPR/Cas9 cut sites (gRNA recognition sequences) are indicated with red arrows. Homology arms and PCR primers used to amplify them from genomic DNA for cloning into the gene targeting construct are shown as violet bars and arrows, respectively. Locations of the PCR primers used to screen for homozygously targeted clones are shown as blue arrows. Endogenous and synthetic promotors are shown as thick arrows. Exons and antibiotic resistance genes are shown as bars. (PDF) [file pgen.1008550.s004.pdf]

Supplementary Figure S4

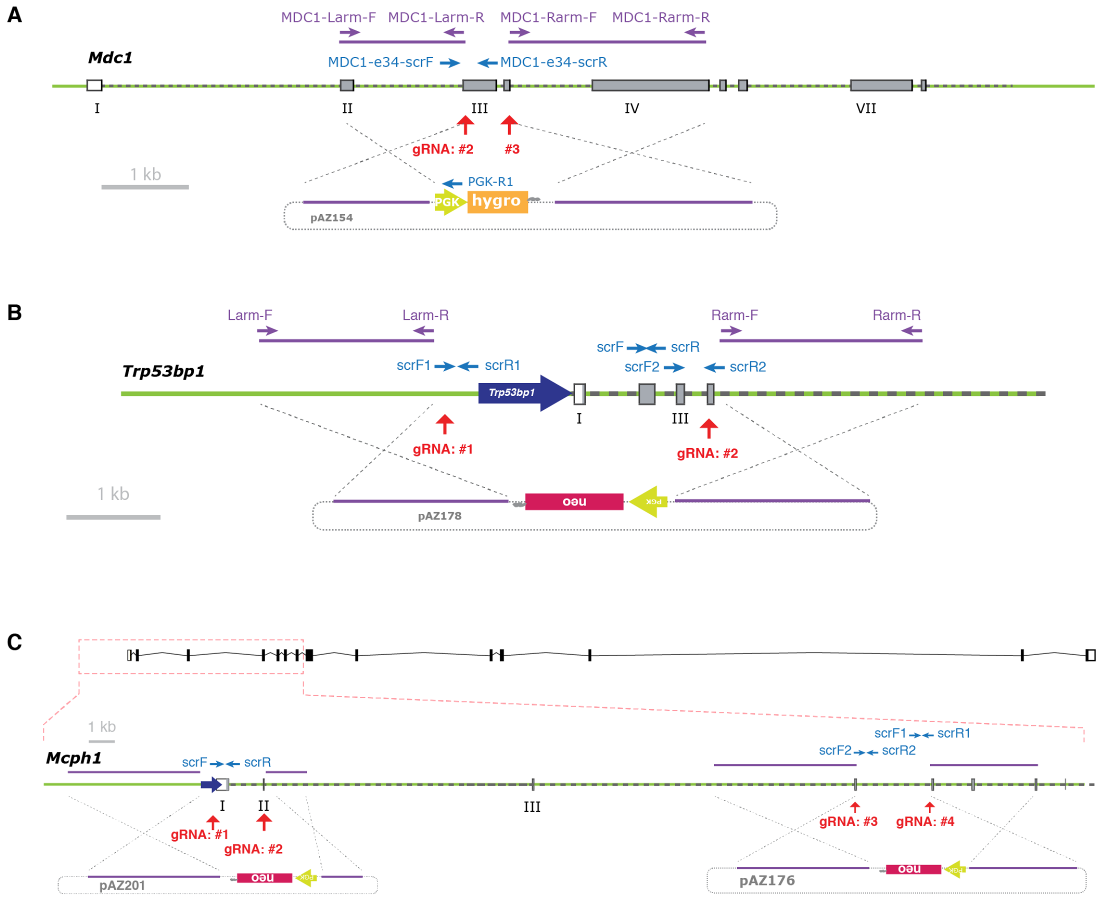

Figure S4 Generation of knock-out mES lines by CRISPR/Cas9-assisted gene targeting Schemes of mouse loci (**A**) *Mdc1,* (**B**) *53bp1* and (**C**) *Mcph1* and of the corresponding gene targeting construct are shown. CRISPR/Cas9 cut sites (gRNA recognition sequences) are indicated with red arrows. Homology arms and PCR primers used to amplify them from genomic DNA for cloning into the gene targeting construct are shown as violet bars and arrows, respectively. Locations of the PCR primers used to screen for homozygously targeted clones are shown as blue arrows. Endogenous and synthetic promotors are shown as thick arrows. Exons and antibiotic resistance genes are shown as bars.
