# Supplementary material for: Low dose ionizing radiation strongly stimulates insertional mutagenesis in a γH2AX dependent manner
Source: PLoS Genet. 2020 Jan 16;16(1):e1008550. doi: 10.1371/journal.pgen.1008550 (PMC6964834; doi:10.1371/journal.pgen.1008550)
Supplement: S5 Fig — Total cell extracts from the mES cell lines with indicated genotypes were fractionated by SDS-PAGE and immunoblotted with the indicated antibodies: (A) anti-MDC1 monoclonal and (B) polyclonal raised against the region encoded by exon 8, (C) anti-53BP1 and (D) anti-MCPH1. Membranes were re-probed with anti-PARP-1 antibody to assess relative loading. For each genotype at least two independent clones used in the experiments were tested. Clones that were derived from the CRISPR/Cas9-assisted gene targeting procedure but retained the wild-type allele (as determined by PCR genotyping) were used as controls in some experiments; these are indicated with letter C. Asterisk indicates non-specific band. (PDF) [file pgen.1008550.s005.pdf]

Supplementary Figure S5

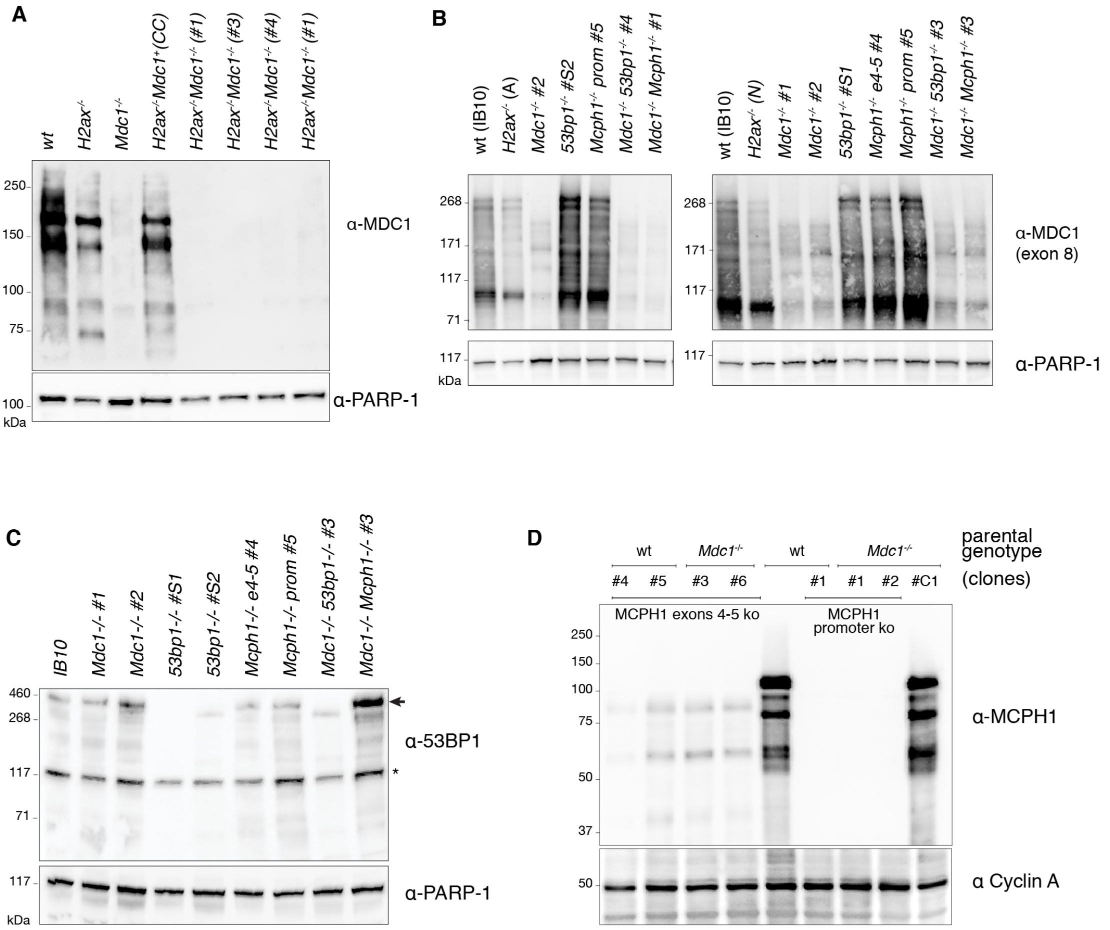

Figure S5 Immunoblots confirming the loss of protein expression from targeted genes Total cell extracts from the mES cell lines with indicated genotypes were fractionated by SDS-PAGE and immunoblotted with the indicated antibodies: (**A**) anti-MDC1 monoclonal and (**B**) polyclonal raised against the region encoded by exon 8, (**C**) anti-53BP1 and (**D**) anti-MCPH1. Membranes were re-probed with anti-PARP-1 antibody to assess relative loading. For each genotype at least two independent clones used in the experiments were tested. Clones that were derived from the CRISPR/Cas9-assisted gene targeting procedure but retained the wild-type allele (as determined by PCR genotyping) were used as controls in some experiments; these are indicated with letter C. Asterisk indicates non-specific band.
