# Supplementary material for: Low dose ionizing radiation strongly stimulates insertional mutagenesis in a γH2AX dependent manner
Source: PLoS Genet. 2020 Jan 16;16(1):e1008550. doi: 10.1371/journal.pgen.1008550 (PMC6964834; doi:10.1371/journal.pgen.1008550)
Supplement: S1 Table — (PDF) [file pgen.1008550.s006.pdf]

S1 Table. S-RI in *Polq Ku70/80* double knock-out ES cells.

		unirradiated			100 mGy		
	number of electroporations	number of cells ($\times10^6$)	plating efficiency	colonies	number of cells ($\times10^6$)	plating efficiency	colonies
***Polq$^{-/-}$Ku70$^{-/-}$* #6-5**	20	67	5.1%	1	133	2.6%	0
***Polq$^{-/-}$Ku70$^{-/-}$* #7-2**	13	43	2.6%	0	87	2.2%	7
***Polq$^{-/-}$Ku70$^{-/-}$* #8-8**	16	62	3.9%	2	98	2.5%	5
***Polq$^{-/-}$Ku80$^{-/-}$* #1**	16	49	5.5%	4	111	2.7%	0
***Polq$^{-/-}$Ku80$^{-/-}$* #3**	24	74	4.7%	1	166	2.6%	0
**total** / *average*	**89**	**295**	*4.36%*	**8**	**595**	*2.52%*	**12**
		**adjusted integration frequency ($\times10^{-6}$):**		**0.62**			**0.80**
