# Supplementary material for: Low dose ionizing radiation strongly stimulates insertional mutagenesis in a γH2AX dependent manner
Source: PLoS Genet. 2020 Jan 16;16(1):e1008550. doi: 10.1371/journal.pgen.1008550 (PMC6964834; doi:10.1371/journal.pgen.1008550)
Supplement: S1 Text — (PDF) [file pgen.1008550.s007.pdf]

Supplementary Text

Estimation of The Frequency with Which DSBs Capture Transfected DNA

Given the exquisite sensitivity of the S-RI to low doses of γ-irradiation, it may appear that the number of DSBs is not sufficient to explain the increase in RI. However, the following calculation suggests that this is not the case. We will use the data from five independent experiments plotted in Figure 1A, and compare unirradiated control with cells irradiated with 10 mGy. In each experiment 6×10^6 ES cells were electroporated with 10 µg circular plasmid DNA and divided over 8 dishes (7.5×10^5 cells/dish). This resulted in an average of 34±4 and 72±14 colonies in plates irradiated with 0 and 10 mGy, respectively (38 additional integrations after 10 mGy).

Frequency of induced DSBs

The widely used number of 35 DSBs per 1 Gy of IR per cell is based on direct (PFGE, doses >10 Gy) and indirect (γH2AX immunofluorescence, doses 0.0012-2 Gy) DSB detection in human fibroblasts (1). Since human and mouse genome are of similar sizes and we do not envision fundamental differences in the effect of ionizing radiation on the DNA in ES cells and fibroblasts, we assume that 10 mGy will induce ~0.35 DSBs per cell or 2.6×10^5 DSBs per dish (7.5×10^5 cells/dish × 0.35 DSB/cell). After correcting for ~50% transfection efficiency, this means that ~10^5 induced DSBs result in 38 induced integration events.

Probability of co-localization of induced DSB and transfected DNA

For integration to occur, the DSB needs to collide with a transfected DNA molecule. Since diffusion of large DNA molecules in the nucleus was found to be undetectably low (2), we can simplify the problem by defining collision as co-localization within a spherical volume ("focus") with an arbitrary radius of 50 nm (corresponds to ~5 nucleosome diameters). The number of transfected DNA molecules in the 50 nm vicinity of a DSB (DNA_{DSB}) is then:

$$DNA_{DSB} = DNA_{nucleus} \times \frac{Focus\ volume}{Nucleus\ volume}$$

Taking 5 µm as average nucleus radius, the ratio between the volume of the focus and the nucleus are the ratio of their radii cubed: $(0.05/5)^3=10^{-6}$.

To estimate the number of transfected DNA molecules in the nucleus ($DNA_{nucleus}$), let us assume that electroporation is 100% efficient and equilibrates the DNA concentration across plasma and nuclear membranes. Then the number of molecules delivered into each nucleus can be calculated:

$$DNA_{nucleus} = \frac{DNA_{cuvette} \times \frac{Total\ nuclei\ volume}{Cuvette\ volume}}{Number\ of\ nuclei}$$

Based on the size of the plasmid (4.4 kbp), average MW of DNA base pair (650 Da/bp) and Avogadro constant, the number of DNA molecules in the electroporation cuvette will be:

$$DNA_{cuvette} = \frac{10^{-5} g}{\left(4.4\ \times 10^3\ bp\ \times\ 650 \frac{g}{mol\ \times\ bp}\right)} \times\ 6\ \times 10^{23}\ mol^{-1} \approx \mathbf{2 \times 10^{12}}$$

The volume of a ⌀10µm nucleus is 523 femtoliter, combined volume of all nuclei will be 0.7% of the 450 µl electroporation cuvette volume:

$$\frac{Vol_{nucleus} \times N_{nuclei}}{Vol_{cuvette}} = \frac{\left(\frac{4}{3} \times \pi \times (5 * 10^{-6}m)^3\right) \times \ 6 \times 10^6}{4.5 \ \times 10^{-7}m^3} = 0.7\%$$

The number of transfected DNA molecules per nucleus is then:

$$DNA_{nucleus} = \frac{2 \times 10^{12} \ \times \ 0.7\%}{6 \times 10^6} \approx \mathbf{2.4 \times 10^3}$$

(or ~10^7 base pairs vs ~7×10^9 bp chromosomal DNA), and the frequency of co-localization between a DSB and a transfected DNA molecule:

$$DNA_{DSB} = 2.4 \times 10^3 \ \times \ \ 10^{-6} = 2.4 \times 10^{-3}$$

Based on these estimations we can estimate the frequency with which induced DSBs that have a transfected DNA molecule in 50 nm vicinity will capture that DNA molecule:

$$\frac{Induced\ integrations}{DSBs\ with\ DNA} = \frac{38}{DNA_{DSB} \times DSB} = \frac{38}{2.4 \times 10^{-3} \ \times \ 10^5} = 0.16$$

This probability of a DSB undergoing mutagenic repair resulting in extrachromosomal DNA insertion is surprisingly high. Furthermore, the calculation is based on the assumption that the transfected DNA molecules are intact and evenly distributed within the nucleoplasm and integrations will be single-copy. But it is well established that most RI events involve multiple copies of transfected DNA, and that transfected DNA molecules actively recombine with each other in the cell and are damaged during the transport to the nucleus (3-5). Moreover, the experiments upon which the calculation is based were performed with circular plasmid DNA, which will need to be linearized before ligation into a DSB; circular DNA integrates with about 10-fold lower absolute efficiency than linearized.

On the other hand, the probability drops precipitously if the focus radius is increased from the arbitrary 50 nm we used: e.g. from 0.16 for 50 nm to 0.02 for 100 nm radius.

1. Rothkamm K, Löbrich M (2003) Evidence for a lack of DNA double-strand break repair in human cells exposed to very low x-ray doses. *Proc Natl Acad Sci U S A* 100(9):5057–5062.

2. Lukacs GL, et al. (2000) Size-dependent DNA mobility in cytoplasm and nucleus. *J Biol Chem* 275(3):1625–1629.

3. Folger KR, Wong EA, Wahl G, Capecchi MR (1982) Patterns of integration of DNA microinjected into cultured mammalian cells: evidence for homologous recombination between injected plasmid DNA molecules. *Mol Cell Biol* 2(11):1372–1387.

4. Chen C, Chasin LA (1998) Cointegration of DNA molecules introduced into mammalian cells by electroporation. *Somat Cell Mol Genet* 24(4):249–256.

5. Würtele H, Little KCE, Chartrand P (2003) Illegitimate DNA integration in mammalian cells. 10(21):1791–1799.
