# Supplementary material for: Low dose ionizing radiation strongly stimulates insertional mutagenesis in a γH2AX dependent manner
Source: PLoS Genet. 2020 Jan 16;16(1):e1008550. doi: 10.1371/journal.pgen.1008550 (PMC6964834; doi:10.1371/journal.pgen.1008550)
Supplement: Attachment — Submitted filename: Response to reviewers.docx [file pgen.1008550.s008.docx]

# Reviewer #1:

This is a review of "PGENETICS-D-19-01201." In this manuscript, the authors examine the mechanism of radiation-induced random integration (RI) of DNA, using very low doses of radiation. These findings are highly significant, as the mechanisms of RI have remained poorly understood, and the results could have implications for medical radiation (see below major point 1). The authors use multiple methods to provide convincing evidence that low levels of radiation induce RI using multiple model systems and radiation sources, as well as the AsiSI nuclease. They also examine the mechanisms of radiation-induced RI. For one they find that loss of major DNA repair proteins do not affect RI, although I am surprised that the Ku-Polq- cell line was not tested (major point 2). In contrast, the H2AX-MDC1 signaling factors are shown to be required for radiation-induced RI, including convincing evidence that the signaling residues on H2AX (S139 and Y142) are required for this function. As the precise roles of H2AX and MDC1 in regulating repair remain unclear, it is understandable that further mechanistic insight has been difficult (although recent studies on how these factors influence NHEJ could be included, Minor point 1), but I think that these findings will stimulate research in this area.

We thank the reviewer for the critical reading of our paper and for the insightful suggestions.

## Major points.

1. There are potential implications of this study for the ability of medical radiation to cause insertion of extra-chromosomal DNA. This issue is raised a few points in the manuscript, and in the experiment using the micro-CT source. However, as all experiments are performed in cell lines, the implications for patients may be unclear. As this has the potential to have effects on public health, and even possible press attention (which has a tendency to over-simplify concerns), these claims should be carefully stated. For instance, the Discussion does not mention these issues in detail, and the caveats of drawing too many conclusion on implications for medical radiation without clinical data.

We agree with this point and modified the abstract, author summary, and the Implications section in the discussion to explicitly state that physiological relevance and public health implications of our *in vitro* findings cannot be understood without further experiments using animal or organoid models and epidemiological studies.

2. Given the recent Nature Communications paper from this group with a Polq-Ku- mESC line, I am surprised that this line is not used to examine possible functional redundancy for radiation-induced RI. Perhaps there are technical issues, e.g. because this line is so radiation sensitive, it may be difficult to separate radiation toxicity vs. RI frequency. Nonetheless, I suggest this issue should be addressed.

We performed the S-RI experiments in Polq–Ku– cells, and report the results in Supplementary S1 Table. Because the double mutant cells are hypersensitive to irradiation and because we expected to need large numbers of cells to observe any possible integration events, we chose a single irradiation dose of 100 mGy. In total we transfected ~ 9 × 10^9^ cells and retrieved very few stably transformed colonies (Supplementary S1 Table). Due to this low frequency of recovered events there is no statistical significance between non-irradiated and irradiated conditions. This is consistent with the results of the Rad54-GFP gene targeting assay used in the previous paper (Figure 3b in our 2017 paper). We therefore left the model unchanged in Fig S3.

### Minor point.

1. Two recent studies indicate that H2AX and MDC1 may suppress precise canonical non-homologous end joining using Cas9 DSBs, which may be considered in the Discussion when addressing potential mechanisms. PMID: 28057860, PMID: 28977657. In other words, both studies indicate that H2AX is important for mutagenic EJ events, and the latter has evidence with MDC1.

We are grateful for pointing out this omission and added the references to the discussion (p 8, line 35).

2. Some discussion early in the Results about the dose of linear DNA introduced into cells relative to the mass of chromosomal DNA should be included, even if it is only an estimated range.

We thank the reviewer for the suggestion. We had actually made such an estimation but did not include in the manuscript. We thought about what the probability would be of a DSB which has a transfected DNA molecule near it to capture this DNA molecule. This was to answer to the question “are there enough DSBs from such low dose?”. We added these calculations as supplementary S1 Text.

# Reviewer #2:

Zelensky et al investigate an observation that ionizing radiation promotes integration of exogenous DNA into mammalian cell genomes. While this observation has been made previously by other groups, Zelensky show that it is most evident at the low doses, and thus plausibly medically relevant (i.e. after medical imaging). More importantly, this group employs a panel of cell lines defective in members of the DNA damage response to investigate the mechanistic basis for this phenomenon. The work describes some surprising and exciting results, and is for the most part technically well performed. It will be of interest to a wide fraction of this journal’s readers. However, a few critical omissions must be corrected.

We are very grateful to the reviewer for the critical assessment of our work and the suggestions on how it can be improved.

## Major omissions.

1) The authors determined in previous work that random integration is ablated in cells deficient in both Ku and Polymerase theta. They appear to infer from this prior result that stimulated RI will be similarly dependent on the presence of at least one of the mammalian end joining pathways (Fig S3C). They must formally confirm this, i.e. show that low level radiation is not sufficient to rescue integration in cells deficient in both Ku and Polymerase theta.

This inference was indeed based on the previous paper, specifically the results of the Rad54-GFP assay we reported in the 2017 paper (Figure 3b), which measures the ratio between random and targeted integration. There we did not observe any effect of irradiation with 200 mGy in Ku and Pol θ deficient cells, and concluded that RI is fully dependent on the two pathways and cannot be forced.

We now tested this conclusion directly, using the S-RI assay, the results are reported in Supplementary S1 Table. Because the double mutant cells are hypersensitive to irradiation and because we expected to need large numbers of cells to observe any possible integration events, we chose a single irradiation dose of 100 mGy. In total we transfected ~ 9 × 10^9^ cells and retrieved very few stably transformed colonies (Supplementary S1 Table). Due to this low frequency of recovered event there is no statistical significance between non-irradiated and irradiated conditions.

2) The authors must confirm efficacy of their checkpoint kinase inhibitor treatments (e.g. using phosphor-specific westerns). This may help resolve apparent contradictory evidence arguing that combined ATRi and ATMi reduced S-RI, even though caffeine treatment, which they suggest inhibits all three kinases, did not. Additionally, the statement “in contrast to UCN-01, caffeine, and wortmannin had no effect on S-RI” appears incorrect. Fig S2E suggests results with UCN-01 are also indistinguishable from untreated.

We added immunoblots showing the effect of inhibitors on phospho-Chk2 and γH2AX (Fig S2F). UCN-01 indeed doesn’t have an effect, and this is what we intended to say in the original sentence, which needed a comma to be grammatically correct. We changed the wording to “In contrast**, treatment with** UCN-01, caffeine and wortmannin had no effect on S-RI” to make the statement clear.

3) The authors routinely quantify stimulated random integration (“S-RI”) using the fraction “RI stimulation”, as defined by the frequency of random integration after stimulation divided by the frequency of random integration without stimulation. It is often not possible to fully interpret such data. For example, there is an apparent dose-dependent increase in S-RI in cNHEJ mutants; does this simply reflect a decrease in the base line (zero dose) integration frequency in the mutants, or is the relationship more complex? Difficult-to-parse statements of interpretation “Genetic analysis of stimulated RI (S-RI) revealed that it is…not reduced by DNA polymerase θ (Polq) inactivation” vs. “Surprisingly, S-RI was increased rather than suppressed in the Pol θ-deficient cells (Polq-/-), and in the Ku mutants” further add to the confusion. Another figure is necessary for at least one of these mutants, in which the authors plot raw random integration frequencies over the full range in dose (0-100mGy). This would also be helpful in interpreting how increasing transfected DNA amounts impact S-RI, as tested in Figure 1H. Again, it would be helpful to see the relationship between raw integration frequencies for e.g. 2 ug vs. 10 ug transfected DNA over the full range in radiation dose. Simply including the “background” RI frequency (Figure 1I) doesn’t provide all the necessary information.

All the data we collect is fully represented by the combination of the background RI plot (N colonies in unirradiated plate / N viable cells plated) and the RI stimulation plot (N colonies in irradiated / N unirradiated), because number of viable cells is not significantly affected by the irradiation doses we work with. We reported this for all experiments, with the exception of the one mentioned by the Reviewer. The reason for the omission is that we included the background RI data from S-RI experiments with NHEJ lines into our 2017 Nature Communications paper. We added an explicit statement about this to the legend of Fig 2B and adjusted to the methods section.

## Minor Concerns

1) The authors leave another significant question unresolved; how long does the irradiation effect last? Regarding Fig 1G, the authors should test a wider range in time.

We performed the suggested experiment, the results are reported as a new panel in Fig 1G. The kinetics of S-RI response is in good agreement with the DSB clearance kinetics determined previously using γH2AX foci quantification and physical methods. We thank the reviewer for this suggestion, because this experiment highlighted the use for the S-RI assay we did not think much about, namely comparing DNA damage persistence under different conditions (repair mutants, types of damage, cell cycle dependence etc.)

2) It is difficult to reconcile results in Figure 2B (RI stimulated 2-3-fold at 100mGy in wild type mES), with results in 2A and 2C (RI stimulated 6-7 fold at 100 mGy in wild type mES).

There indeed can be substantial variation in the exact magnitude of the effect between experiments. This is not surprising to us, given the high sensitivity of the assay to damage induction and strong dependence on parameters such as amount of DNA. The plots in figure panels represent experiments in which cell lines with different genotypes were analyzed side by side to determine the effect of the genotype.

3) The distinction between low (P7 gate) and high (P4 gate) GFP expression in Fig S1C should either be rationalized or omitted.

We added the sentence to the legend to state that the low gate (P7) better reflects the transient transduction efficiency, while the more conservative gate (P4) is used to more stringently identify the population of cells where GFP is still continuously produced due to viral integration, rather than remains rom the transiently transduced state.

4) The statement that “wortmannin that has highest specificity for DNA-PKcs” should be more carefully worded, in that it could be interpreted as intending to state that “amongst DNA-PKcs inhibitors, wortmannin has the highest specificity”. There are other DNA-PKcs inhibitors that are more specific for DNA-PKcs.

Thank you for pointing this out. We adjusted the text to clarify this (p7 lines 3-4).

# Reviewer #3:

The report by Zelensky and co-workers provides convincing evidence that insertional mutations can occur following radiation doses that are in the range of medical imaging modalities. They also show that the process is more efficient at low extrachromosomal DNA levels and that radiation-induced (or simulated) random insertions may be mechanistically distinct from background (un-stimulated) radiation insertions. The experimental measurements were conducted at relatively high dose rates. It would have been interesting if the authors had examined dose rate as a variable in the induction of S-RI given that the preclinical CT dose response relationship showed a plateau while the higher dose rate Cs-137 irradiator did not. The results challenge conventional thinking regarding low dose effects and suggest that further study will be important to understand the implications of the work regarding the risks of radiation-induced detriment following medical imaging. As the authors acknowledge these observations would need to be demonstrated in a pre-clinical intact animal model, a much more difficult proposition, to impact current understanding of low dose effects. It is particularly important that the authors emphasize this to avoid having this work be misconstrued by other scientists or the general public.

We thank the reviewer for appreciating our findings.

We agree that further experiments are required and modified the abstract, author summary and the “Implications” section in the discussion to stress this point.

We cannot readily perform the experiments with lower dose rates using the equipment we currently have access to. In the initial stages of the project we compared the effect of irradiations using Cs-137 source with and without a 75% metal attenuator, and did not see a difference. However, the difference in the dose rate may not have been high enough.
